# Modern Management of the Exstrophy-Epispadias Complex

**DOI:** 10.1155/2014/587064

**Published:** 2014-01-05

**Authors:** Brian M. Inouye, Ali Tourchi, Heather N. Di Carlo, Ezekiel E. Young, John P. Gearhart

**Affiliations:** Division of Pediatric Urology, Department of Urology, James Buchanan Brady Urological Institute, Charlotte Bloomberg Children's Hospital, The Johns Hopkins University School of Medicine, 1800 Orleans Street, Suite 7302, Baltimore, MD 21287, USA

## Abstract

The exstrophy-epispadias complex is a rare spectrum of malformations affecting the genitourinary system, anterior abdominal wall, and pelvis. Historically, surgical outcomes were poor in patients with classic bladder exstrophy and cloacal exstrophy, the two more severe presentations. However, modern techniques to repair epispadias, classic bladder exstrophy, and cloacal exstrophy have increased the success of achieving urinary continence, satisfactory cosmesis, and quality of life. Unfortunately, these procedures are not without their own complications. This review provides readers with an overview of the management of the exstrophy-epispadias complex and potential surgical complications.

## 1. Introduction

**Figure 1:**
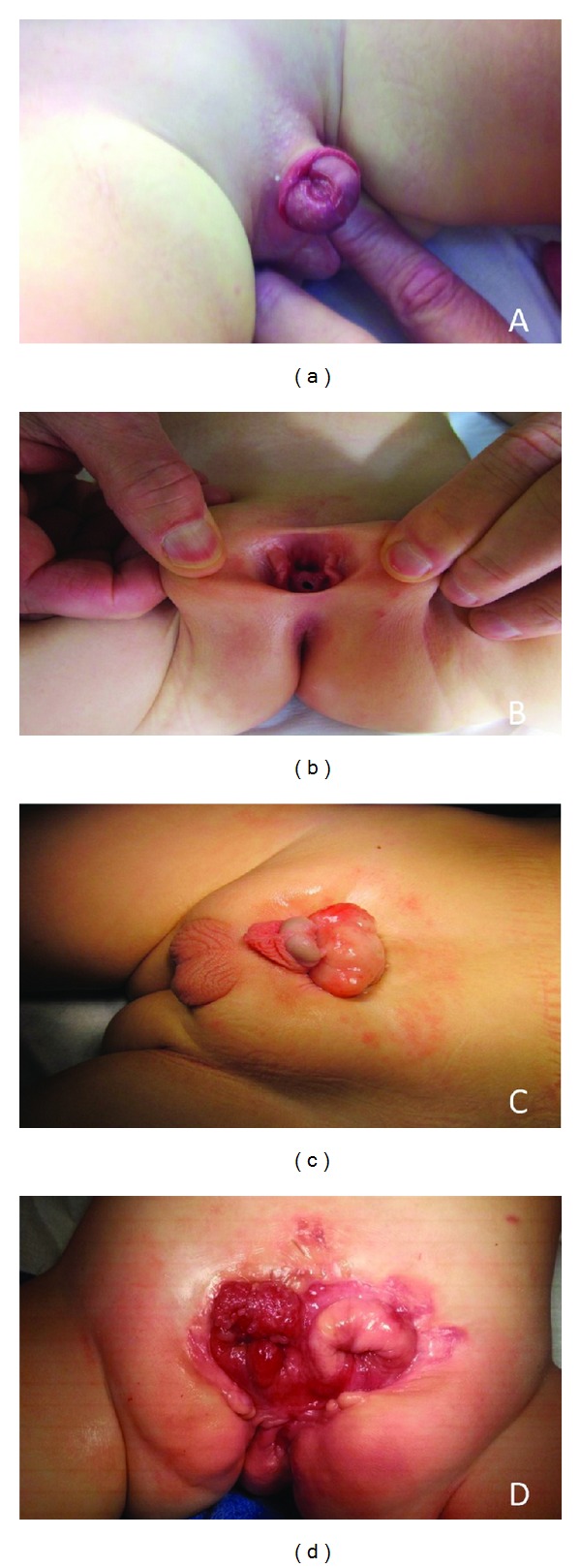
Four presentations of the exstrophy-epispadias complex: (a) complete male epispadias, (b) complete female epispadias, (c) classic bladder exstrophy, and (d) cloacal exstrophy.

The exstrophy-epispadias complex (EEC) is a rare spectrum of defects affecting the genitourinary and gastrointestinal tracts, musculoskeletal system, pelvic floor musculature, and bony pelvis. The three most common presentations of EEC are epispadias, classic bladder exstrophy (CBE), and cloacal exstrophy (CE) (Figure 1). Complete epispadias is the least severe form of EEC and presents with a dorsally open urethral meatus with mild pubic diastasis and a closed anterior abdominal wall and bladder. CBE, the most common presentation of EEC, presents with a wide pubic diastasis and an abdominal wall defect exposing an open bladder and urethra with an epispadiac opening. CE, the most severe of the three presentations, is similar, but a portion of cecum or hindgut separates the two open hemibladders. CE also presents with malformations of the gastrointestinal, musculoskeletal, and central nervous systems, also known as the OEIS (omphalocele, exstrophy, imperforate anus, and spinal abnormalities) complex.

Most patients with EEC undergo multiple reconstructive surgeries beginning with closure of the bony pelvis, bladder, and anterior abdominal wall, followed later by epispadias repair. Oftentimes, children with CBE and CE must undergo pelvic osteotomy and lower extremity immobilization to ensure complete approximation and sufficient deepening of the pelvis for anatomic placement of the bladder. While current techniques achieve reasonable success in preservation of renal function, continence, and cosmesis, there are also many recognized complications associated with reconstruction.

## 2. Epidemiology

Complete epispadias is a rare congenital malformation. It occurs in one in every 117,000 male births, and only one of every 484,000 female births [1]. CBE is the most common presentation of EEC, occurring in approximately one per 10,000 to 50,000 births [2] and affecting males approximately twice as often as females [3]. Risk factors include Caucasian race, young maternal age, and maternal multiparity [4]. Furthermore, EEC is increased among children conceived with assisted-reproductive technologies such as in vitro fertilization [5]. Arising in one in 200,000 births, CE is a more rare and severe presentation of EEC. Out of an institutional review board-approved database of 1202 EEC patients, only 112 patients had CE. Improved prenatal diagnosis increases the detection rate, allowing for elective termination of pregnancy. CE appears to be as much as twice as common among males as females. No risk factors have been definitively associated with an increased risk of CE [6].

## 3. Etiopathogenesis

While the cause of EEC is not completely understood, it is theorized to result from a disorder of cloacal membrane development. During the fourth gestational week, the cloacal membrane may overdevelop, preventing mesenchymal migration between the ectoderm and endoderm. It is thought that this malformation not only inhibits normal development of the lower abdominal musculature and pelvic bones, but also makes the cloacal membrane unstable and prone to early rupture. The timing and location of rupture of the cloacal membrane dictate the patient's presentation along the exstrophy-epispadias spectrum [7, 8]. Epispadias occurs if the rupture produces a division or nonunion at the distal end of the urinary tract. CBE results if the rupture occurs after the urorectal septum divides the gastrointestinal from the genitourinary tracts while CE results if the rupture occurs before this separation [9].

There has also been a growing understanding of the molecular and genetic etiology of EEC. p63 is a member of the p53 tumor suppressor family that is highly expressed in stratified epithelium including the bladder and its overlying skin [10]. Its expression is decreased in CBE patients compared to controls, and p63 knockout mice have CBE-like anomalies [11, 12]. These results led to the recent finding that insertion and deletion polymorphisms of ΔNp63 lead to the reduced p63 expression that may cause EEC [13].

## 4. Functional Anatomy and Associated Anomalies of the Exstrophy-Epispadias Complex

### 4.1. Urogenital Anomalies

While the bladder is normal in epispadias, it is exposed anteriorly through the abdominal wall in both CBE and CE. In most cases the bladder and abdominal wall should be closed soon after birth. However, if the bladder template is too small (<3 cm), is covered with polyps, or appears inelastic [14, 15], then primary closure should be delayed. Histologically, the exstrophic bladder appears immature, demonstrating significantly fewer myelinated nerves per field as compared to controls; however, there is potential for normal development after a successful initial closure [16]. If the patient's bladder capacity does not increase sufficiently following closure, the patient may ultimately need augmentation cystoplasty (AC) [17]. In cases when the bladder is excessively fibrotic or is too small even to attempt augmentation, then bladder substitution surgery, in the form of orthotopic neobladder or a continent catheterizable pouch, is generally undertaken [18].

The ureters in CBE and CE patients enter the bladder at an abnormal angle leading to vesicoureteral reflux (VUR) in all patients following bladder closure [19, 20]. As long as VUR does not lead to upper urinary tract changes, the ureters are most commonly reimplanted into the bladder at the time of augmentation or bladder neck reconstruction in staged repair. They can also be safely reimplanted during primary complete repair [21]. Independent of changes secondary to VUR, the upper urinary tract is malformed in about one-third of all EEC cases with the highest frequency occurring in CE patients. Common anomalies include ureteropelvic junction obstruction, horseshoe kidney, and ectopic kidney [22].

Males with EEC all have a urethral meatus located dorsally between the penopubic angle and the proximal glans. Distal to its ectopic opening, the urethra is open dorsally creating a spade-like appearance. A schematic of this male epispadias can be found in *The Netter Collection of Medical Illustrations* [23]. Compared to controls, the phallus is also shorter and broader often with significant dorsal chordee. Contributing to this foreshortened appearance is the lateral displacement of the corporal bodies under the pubic bones [24]. In CE, the phallus is typically split completely between the diastatic pubis, with each half often of unequal size [25].

In EEC females, the distal aspect of the dorsal urethra remains open, resulting in a patulous bladder neck. The mons pubis is flattened and displaced laterally while the vagina and introitus are displaced anteriorly. The bifid clitoris is usually located in the anterior vaginal wall which is surrounded by divergent labia. The vagina is often short or stenotic [26]. Mullerian anomalies are common in female CE patients and include vaginal or uterine duplication or sometimes complete agenesis [22].

### 4.2. Musculoskeletal Anomalies

While the anterior abdominal wall is intact in epispadias, in CBE and CE, the bladder and urethra are exposed through a triangular defect in the lower abdominal wall. The opening extends from the umbilicus to the intrasymphyseal band inferiorly. Umbilical hernias are common but are usually insignificant and can be repaired at the time of primary closure. Indirect inguinal hernias, due to a persistent processus vaginalis, large inguinal rings, and the relatively straight direction of the inguinal canal are common in CBE and CE and are also easily repaired [27].

Patients with EEC often demonstrate diastasis of their pubic rami with divergent distal rectus abdominis muscles. Compared to controls, CBE patients have a mean pubic diastasis of 4.8 cm, external rotation of both the anterior and posterior segments of the pelvis, 30% shortening of the anterior pelvis, increased distance between the triradiate cartilage, and retroversion of the acetabulum [28]. These patients also have wider sacroiliac joint angles, a more inferiorly rotated pelvis, and a larger sacrum when compared to nonexstrophy patients [29]. While bone segments in CE pelvises are similar in length, there are more malrotation and asymmetry than in CBE pelves [22, 28, 30]. These pelvic deformities may cause the child to ambulate with a waddling gait.

Magnetic resonance imaging (MRI) studies have shown that the pelvic floor musculature is also significantly different in preoperative EEC patients. Compared to controls or postoperative patients, the levator ani in CBE patients has a larger mean area, is located more posteriorly to the rectum, and is externally rotated and flattened resulting in a “box-like, open book” pelvis with an anteriorly positioned bladder [31]. The obturator internus and externus are also both outwardly rotated [32]. As with the bony pelvis abnormalities, CE patients have similar, but more severe, pelvic floor musculature abnormalities than CBE patients. These deficits contribute to incontinence in these patients and predispose females to uterine prolapse [33].

### 4.3. Gastrointestinal Abnormalities

While epispadias patients do not have associated gastrointestinal anomalies, patients with CBE and CE have an anteriorly displaced anus and anal sphincter. In combination with the aforementioned pelvic floor muscular deficits, this misaligned anal sphincter predisposes exstrophy patients to fecal incontinence. While CBE patients occasionally have omphalocele, imperforate anus, rectal stenosis, and rectal prolapse [34], CE patients almost always have some gastrointestinal defect. These include omphalocele, imperforate anus, rudimentary hindgut, malrotation of the bowel, and short gut syndrome, the last of which is often compounded by multiple bowel surgeries [6, 25, 35]. The rudimentary hindgut, or a portion of duplicated cecum, can also be found separating the CE patient's two bladder plates [25].

### 4.4. Neurospinal Abnormalities

Neurospinal defects are absent in epispadias patients. Approximately 7% of CBE patients will have a spinal abnormality such as spina bifida occulta, scoliosis, and hemivertebrae. Most of the abnormalities are uncomplicated, but spinal dysraphism may cause neurologic dysfunction [36]. Nearly all CE patients demonstrate significant neurospinal deficits including neural tube defects, vertebral anomalies, spinal myelodysplasia, spinal dysraphism, and tethered cord [37]. These associated anomalies necessitate prompt neurological evaluation with spinal US and MRI and can further exacerbate urinary and bowel incontinence, lower extremity immobility, and erectile dysfunction [38, 39].

## 5. Diagnosis

The majority of EEC cases are first noted on postnatal exam. However, CBE and CE can be diagnosed prenatally with fetal transabdominal ultrasound (US) between the 15th and 32nd weeks of pregnancy [22]. Antenatal imaging demonstrating absence of bladder filling, a low-set umbilicus, widened pubic rami, small genitalia, and a lower abdominal mass that increases throughout the duration of pregnancy may indicate CBE or CE [40, 41]. Furthermore, the prolapsed ileum in CE patients may look like an “elephant trunk” on US [42]. In the approximately 25% of exstrophy cases that are diagnosed prenatally [43] delivery should be arranged at a specialized medical center with expertise in managing this complex anomaly. Similarly, infants who are diagnosed at birth should be promptly transported to such centers to allow for an experienced evaluation and possibly primary closure [44].

## 6. Male Epispadias Repair

Epispadias repair includes correction of dorsal chordee, glanular and urethral reconstruction, and closure of penile skin [45]. The modern modified Cantwell-Ransley repair advances the urethral meatus to an orthotopic position utilizing a reverse meatal advancement and glanuloplasty technique [46]. The dorsal chordee is released by mobilizing the urethral plate from the underlying corpora from the level of glans down to the prostatic urethra. The corporal bodies are anastomosed at the dorsal medial aspect over the tubularized urethra. Older patients may have persistent chordee, in which case a cavernostomy may be required [47]. Outcomes are significantly impacted by the quality and quantity of the penile skin and urethral plate available, which tend to decrease with each subsequent attempt at repair. Intramuscular or topical testosterone may be utilized preoperatively to improve the quality and quantity of penile skin and thereby minimize the risk of fistulae or other complications [48].

Mitchell and Bagli have described a further modification of Cantwell-Ransley repair in which the urethral plate and each corporeal body along with its hemiglans are dissected completely free from each other. The urethra is then tubularized and placed into an anatomic, ventral position [49]. It is felt that this “complete penile disassembly” can be performed safely because each of the corpora has completely separate blood supply in the epispadiac phallus. However, because the urethral plate draws its blood from the spongiosa, complete disassembly may be ischemic. This method of epispadias repair is often performed at the time of primary bladder closure, the combination of which is called “complete primary repair of bladder exstrophy” (CPRE). When performed as part of CPRE the complete penile disassembly technique requires more extensive proximal mobilization in order to place the bladder deeply into the pelvis. In addition, it should be noted that the lateral dissection during this procedure can result in neurovascular bundle injury and consequent erectile dysfunction [50]. When it is possible to place the bladder into a deep orthotopic position during CPRE, the tubularized urethra is usually shorter than the actual corpora, resulting in hypospadias and necessitating further surgical repair and the attendant risk of further associated complications [51].

Following epispadias repair, patients receive yearly gravity cystograms to measure bladder capacity. When the patient desires continence (typically 5 to 9 years of age), he will undergo a continence procedure, such as a Young-Dees-Leadbetter bladder neck reconstruction (BNR), if the bladder capacity is sufficient. If the bladder template is too small, the patient will instead undergo a combination of a bladder neck transection, bladder augmentation, and continent urinary diversion.

## 7. Female Epispadias Repair

Due to the comparatively shorter urethra, repair of isolated female epispadias is generally done along with Young-Dees-Leadbetter bladder neck reconstruction (BNR), monsplasty, and clitoroplasty. Like male epispadias patients, if the bladder template does not grow after BNR, the patient may require bladder neck transection, bladder augmentation, and continent urinary diversion. As these patients also have a pubic diastasis, bilateral iliac osteotomies may be necessary [52].

## 8. Classic Bladder Exstrophy Repair

Historically, exstrophy patients were treated with cystectomy and often died at a young age secondary to complications of renal failure. Current treatments preserve the bladder in nearly every patient and allow for continence through the urethra in most [53, 54]. However, if the primary closure fails or if the patient's bladder remains small or noncompliant it may require AC with or without a continent urinary diversion (CUD) to achieve dryness [55–57]. These patients will need to perform clean intermittent catheterization (CIC) to empty their bladders/pouches.

Repair of bladder exstrophy begins with closure of the bladder and abdominal wall by either the modern staged repair of exstrophy (MSRE) or CPRE. Pelvic osteotomies may be performed at the time of primary closure in order to deepen their flattened pelvis, close the pubic diastasis, and release tension on the abdominal wall. Successful primary closure is of utmost importance since it is associated with decreased overall costs, decreased inflammation and fibrosis of the bladder, improved bladder growth, and decreased need for urinary diversion [15, 58–62].

There is a debate over the timing of the primary closure with proponents of early bladder closure (closure during the first 72 hours of life) arguing that prompt closure allows for earlier bladder cycling, improved bladder expansion, and decreased risk of precancerous changes [63]. Those delaying bladder closure state that it does not cause metaplastic changes, can allow for concomitant epispadias repair, and increases the likelihood of postclosure bladder growth in the case of a smaller template [64].

### 8.1. Modern Staged Repair of Bladder Exstrophy (MSRE)

Operative details of the MSRE procedure can be found in *Pediatric Urology *[65]. Seventy percent of patients who undergo MSRE achieve dryness with minimal complications [54]. The first stage is the abdominal wall and bladder closure [66]. Females also undergo genitoplasty and urethroplasty with this first procedure. As previously stated, this stage may be delayed if the bladder template is too small or covered with polyps [67]. The second stage in males is to close the urethral epispadias with a modified Cantwell-Ransley repair at 6 to 12 months of age, if their urethral groove is of adequate length. Following epispadias repair, the patient's bladder capacity is measured annually with gravity cystogram under anesthesia.

To allow for proper bladder growth, the third MSRE stage, a continence procedure such as the Young-Dees-Leadbetter BNR, is delayed until the patient achieves a bladder with adequate capacity and desires continence (usually between 5 and 9 years of age). This stage is combined with ureteral reimplantation to repair VUR. Children who are not candidates for BNR or who fail to achieve urinary continence after the procedure may require bladder neck transection, AC, and continent catheterizable stoma.

### 8.2. Complete Primary Repair of Exstrophy (CPRE)

Unlike MSRE, CPRE combines primary abdominal wall and bladder closure with epispadias repair and partial tightening of the bladder neck [68]. Furthermore, bilateral ureteral reimplantation can safely be done during this surgery to reduce the risk of future febrile urinary tract infection and hydronephrosis [21]. Operative details of the CPRE procedure can be found in *Pediatric Urology *[65].

Proponents argue that this technique may decrease costs, decrease the morbidity associated with multiple operations, and stimulate early bladder growth. The epispadias repair is done by “penile disassembly,” where the urethral plate is fully dissected from the corporal bodies. This technique facilitates urethral closure and positions the bladder neck posteriorly into the pelvis [49]. While CPRE is purported to reduce the number of surgeries for CBE, many children still require surgery for resulting hypospadias, persistent vesicoureteral reflux, incontinence, or failed primary closure [68, 69]. Potential advantages of CPRE over MSRE may require longer follow-up with additional cases, although the rarity of EEC makes this a significant challenge.

### 8.3. Pelvic Osteotomies and Immobilization

Pelvic osteotomies are recommended in patients who no longer have a malleable pelvis, which usually occurs after 72 hours of age. Osteotomies may increase surgery time and risk for postoperative complications. However, the use of osteotomies during closure is associated with improved success of primary closure by providing a tension-free approximation of the pubic symphysis and abdominal wall that also results in deeper placement of the bladder into the pelvis [70].

A combination of bilateral anterior transverse innominate and vertical posterior iliac osteotomies has been shown to decrease the rate of abdominal dehiscence and bladder prolapse as compared to other osteotomies [71]. At the time of osteotomy, fixator pins and external fixation devices can be placed and left postoperatively for 4 to 6 weeks as the patient is immobilized by one of several described methods. Modified Buck's traction exerts pull longitudinally on the lower extremities and is used after osteotomy. Modified Bryant's traction, where the hips are placed into 90 degrees of flexion, may be used if there is no osteotomy. Spica casts also immobilize the pelvis without the need for external fixators or traction [72]. However, these casts, along with the technique of “mummy wrapping” the child's legs, have been called into question after a retrospective study.found them to cause higher rates of skin breakdown and have lower success rates compared to patients who were placed in modified Buck's or Bryant's traction [70]. The fixators and pins can be surgically removed when good callous formation is seen on pelvic radiography, which usually occurs at 6 to 8 weeks postoperatively.

### 8.4. Bladder Augmentation

After having failed one attempted CBE closure, the chance of achieving adequate bladder capacity for a BNR and continent urethral voiding, decreases to 60% [73]. A bladder that is noncompliant or of insufficient capacity may undergo AC [74]. Common techniques utilize segments of bowel, stomach, or redundant ureter to expand the bladder wall.

### 8.5. Continent Urinary Diversion

CUD is typically required when a patient undergoes AC. A segment of appendix or ileum may be utilized to connect the bladder to the skin and provide a continent stoma through which to perform CIC [75].

## 9. Management of Cloacal Exstrophy

Prior to the 1960s, CE was considered a fatal congenital anomaly with most who survived gestation later dying in the neonatal period [76, 77]. Since the advancement of surgical techniques, survival rates have risen to nearly 100%. Like CBE, the surgical management of CE includes osteotomy and immobilization, bladder and abdominal wall closure, an antireflux procedure, and usually AC with CUD. Due to its many associated anomalies CE requires more arduous pre- and postclosure management.

### 9.1. Preclosure Management and Procedures

After delivery of the child, the exposed bladder and bowel mucosa should be covered or wrapped to avoid mechanical irritation. The high incidence of neurologic anomalies in CE patients requires a spinal ultrasound or MRI along with a neurology or neurosurgery consultation within the first 48–72 hours of life [38]. Furthermore, most CE patients have rudimentary hindgut proximal to an imperforate anus. Intestinal diversion is necessary in such cases and is performed along with omphalocele repair by a general pediatric surgeon. Because these children are at risk for short gut syndrome, it is important to preserve any rudimentary hindgut and to not discard any bowel, as this can be used for reconstruction later in life. The general surgeon should be joined by a pediatric urologist who can approximate the posterior aspect of the exposed bladder halves.

Gender assignment is a crucial conversation that surgeons need to have with parents of CE patients. Many males have an unreconstructable, diminutive phallus and undescended testicles. The parents of these patients may elect to have their son undergo a gonadectomy and be raised as a female. Studies report conflicting conclusions on the possible psychosocial and behavioral outcomes of genotypic males being raised as females given the concept of androgen imprinting [78–80]. Due to the advancement in phallic reconstruction, many are advocating for assigning gender that is consistent with karyotype [6]. If not undergoing gender reassignment, CE males should undergo orchidopexy to preserve testicular histology [81].

### 9.2. Abdominal Wall and Bladder Closure

While osteotomy is not always necessary for CBE, CE patients require bilateral iliac osteotomies due to their wide pubic diastases [82]. In a one-stage CE closure, the osteotomies and bladder closure are performed with omphalocele closure and intestinal diversion within the first 48–72 hours of life [68, 76]. If the infant does not have many associated anomalies, then closure of the two bladder halves and genital revision can also be included during this initial repair. Alternatively, a two-staged approach allows 6 months to pass after omphalocele repair and intestinal diversion before the patient undergoes osteotomy and then, three weeks later, bladder closure and genital revision. Such a delay is indicated if the neonate has many associated anomalies, is not medically tolerating the first parts of the surgery (the omphalocele closure), has a small bladder template preoperatively, or has a pubic diastasis that is too large [83].

### 9.3. Postclosure Management and Procedures

Like CBE patients, CE patients are kept immobilized postoperatively for 4–6 weeks to allow the osteotomies to heal. Also similar to CBE repair, if phallic reconstruction was not performed at the time of initial repair, CE patients then undergo a modified Cantwell-Ransley repair of epispadias at age 1 once their urethral groove is of adequate length [6]. Following epispadias repair, the patient's bladder capacity is measured annually with gravity cystogram under anesthesia.

The majority of CE patients require an AC and continent catheterizable stoma or a CUD at 6 to 8 years of age to achieve continence. During this procedure the patient will either undergo a Young-Dees-Leadbetter BNR or bladder neck transection along with ureteral reimplantation to prevent further VUR [6].

Male patients, who continue to be raised as males, may require a phalloplasty or neophallus if their original phallus is demure. Radial forearm free flaps have been used to reconstruct the phallus. During such a repair, the native glans and ejaculatory ducts are left at the ventral surface of the proximal neophallus in case of failure. They can both be buried into the base of the neophallus after three months, at which point the original procedure is considered successful [84]. Patients may elect to have an inflatable penile prosthesis (IPP) placed a year after the original surgery to allow for erections.

## 10. Prognosis 

Seventy-five percent of male epispadias patients are incontinent, but 80% are continent postoperatively [1]. Those who are still incontinent may require later BNR [22]. Although there is a paucity of literature on isolated female epispadias, reports suggest 87% to 100% continence after repair [52].

In CBE and CE, the single most important predictor of long-term bladder growth and continence is successful primary bladder closure [55, 60]. Continence in CBE children after primary closure ranges from 80 to 100% [6]; however, the chance of achieving an adequate bladder capacity decreases to 60% with primary closure failure and to 40% with a second failure. Furthermore, with the decreased chance of sufficient bladder capacity there is only a 20% chance of becoming continent after a second failed closure [59]. While CPRE may decrease the number of operations to achieve continence by increasing bladder outlet resistance at an early age, many patients still require BNR [85]. Furthermore, only 20–56% of CPRE patients who remain incontinent achieved continence following additional BNR [86, 87].

Wound dehiscence, bladder prolapse, bladder outlet obstruction, and formation of a vesicocutaneous fistula are all outcomes that should be considered failed closures, and each requires subsequent repair [88, 89]. Even though pelvic osteotomy and postoperative immobilization extend the patient's hospital stay up to a month, they have shown to decrease the rate of failure of redo repairs in carefully selected patients [70, 90].

## 11. Potential Complications

The most common complications of both Cantwell-Ransley and complete penile disassembly epispadias repair are persistent chordee, urethrocutaneous fistula, and wound dehiscence [49, 91, 92]. A short urethral plate has been seen in both repairs but is more common with the Mitchell repair [51]. A complication specific to the Mitchell repair's complete disassembly technique is glans and/or corporeal ischemia. Although the exact mechanism of ischemic changes is unknown, it may be secondary to venous congestion, unintentional injury, or abnormal blood supply and collaterals that prevent sufficient blood flow during the procedure [93].

Both single and staged repairs have been shown to be suitable procedures to repair CBE and CE. Wound dehiscence, bladder prolapse, bladder outlet obstruction, and vesicocutaneous fistula formation that require reclosure have been reported with both techniques. Additional intestinal complications such as ileus, volvulus, and small bowel obstruction can occur. Like the Mitchell epispadias repair, CPRE specifically can also lead to the significant complication of penile loss following penile disassembly [6, 87, 88, 93].

Osteotomies increase the risk of transient nerve and muscle palsies (which typically resolve by 12 weeks post-op), delayed ileal union, superficial infection, and inflammation at pin sites. Furthermore, osteotomies may fail, leading to a recurrent pubic diastasis, if the pelvis partially rotates or if there is continued delayed of growth of the ischiopubic bone [94].

AC and CUD also present unique potential complications such as bladder calculi, chronic bacterial colonization, epithelial polyps, and mucus overproduction [73]. Individually, AC may also lead to metabolic acidosis and carcinoma [74], while the stoma created on the skin for a CUD has been associated with stomal complications such as stenosis, prolapse, ischemia, and leakage [75].

Phalloplasty complications include partial necrosis, flap loss, and need for anastomotic revision [84]. When an IPP has also been placed further potential complications include infection and erosion, both of which require explantation. Fortunately, this has become less common with the advent of antibiotic-coated prostheses [95].

## 12. Conclusion 

In the past, CBE and CE were devastating multisystem birth defects with drastic negative impacts on the full range of patient's genital and urinary function. However, using current methods of bladder closure, pelvic osteotomies, and traction and immobilization the majority of these patients can obtain full continence, adequate sexual function, and much overall improvement in quality of life. These advancements also come with a unique spectrum of complications that all physicians involved in their care should be aware of.

## Abbreviations

EEC: Exstrophy-epispadias complex
CBE: Classic bladder exstrophy
CE: Cloacal exstrophy
OEIS: Omphalocele, exstrophy, imperforate anus, and spinal abnormalities
VUR: Vesicoureteral reflux
AC: Augmentation cystoplasty
MRI: Magnetic resonance imaging
US: Ultrasound
CPRE: Complete primary repair of exstrophy
BNR: Bladder neck reconstruction
CUD: Continent urinary diversion
MSRE: Modern staged repair of exstrophy
CIC: Clean intermittent catheterization.
